# Assessing the impact of tech-driven solutions to manage COVID-19 in Saudi Arabia: insights from health information technology professionals

**DOI:** 10.3389/fpubh.2024.1413263

**Published:** 2024-12-19

**Authors:** Nesren S. Farhah

**Affiliations:** Department of Health Informatics, College of Health Science, Saudi Electronic University, Riyadh, Saudi Arabia

**Keywords:** health information technology, e-health, COVID-19, public health, risk health

## Abstract

**Introduction:**

The outbreak of the COVID-19 pandemic has disrupted the provision of healthcare services, forcing health systems, governments, and other public health stakeholders to embark on alternative means to deliver care.

**Method:**

This scenario has fueled the adoption of digital health technologies in the healthcare industry to enhance the management of the COVID-19 pandemic. This study aimed to investigate the effect of using health information technologies (HITs) in managing the COVID-19 pandemic in Saudi Arabia from the health information technology professionals' perspectives. In addition, the study aimed to evaluate the usefulness of using HITs interventions to manage COVID-19, assess HITs information exchange capability and quality, and measure practitioners' satisfaction with HITs.

**Results:**

The present study used a primary analysis and cross-sectional design. Data were collected using survey questionnaires and analyzed. A total of 371 participants (*n* = 371) responded to the questionnaire that was administered online, in March 2022. The study found that HITs significantly influenced the management of the COVID-19 pandemic in various areas, including consulting, screening, and monitoring. However, technical issues, among other challenges, hindered the realization of the desired outcomes.

**Discussion:**

In addition, the study found HITs helpful in managing COVID-19, adding that these technologies improved providers' satisfaction concerning access to and value of information. The study found the need to address the challenges associated with HITs that prevent the effective deployment of HITs to manage COVID-19 and future pandemics.

## 1 Introduction

The outbreak of the coronavirus disease (COVID-19) in early 2020 posed a significant challenge to global health systems. According to Malden et al., COVID-19 led to over 100 million infections and ~2.3 million deaths by February 2021 (1). Following the outbreak of the health pandemic, governments across the world implemented stringent measures, such as lockdowns and social distancing mandates, to counter the spread of COVID-19 infections. However, despite these unprecedented measures, Malden et al. emphasized that a long-term solution to the global health pandemic entailed producing vaccines and effective treatments (1). In addition, the authors argued that some scholars and providers believed that health information technology (HIT), such as electronic health records (EHRs), could play an essential role in bringing the outbreak under control (1). For example, in China, the health system and public health stakeholders leveraged HIT for monitoring, detecting, issuing warnings, preventing, and controlling the outbreak (2). HIT constitutes different information and communication technologies, such as telehealth and remote monitoring devices, used in collecting, transmitting, displaying, and storing patient data (3, 4). Thus, the COVID-19 pandemic was the primary catalyst for promoting HIT adoption in many health systems globally.

During the COVID-19 pandemic, the use of HIT to contain COVID-19 infections increased in the Middle East. Hassounah et al. claim that based on the lessons learned from the global experiences with the 2009 H1NI pandemic, the Middle East launched a GPS-based risk evaluation tool and used Google Maps to represent COVID-19 cases in the region and the world geographically (5). The Kingdom of Saudi Arabia was among the many nations that proactively implemented disease containment measures to restore people's health and wellbeing and end the pandemic. According to Alghamdi et al., Saudi Arabia's Ministry of Health (MOH) launched digital health platforms (DHTPs) that offered follow-up care and monitoring services related to COVID-19 outbreaks. DHTPs enable information communication technologies (ICTs) to offer health services remotely and support decision-making processes for health experts (6). In addition, other health technologies, such as artificial intelligence (AI), Machine Learning (ML), and mobile health applications, enable healthcare professionals and relevant stakeholders to screen people in large gatherings, predict infection rates, monitor the spread of infections, and attain faster diagnosis (6). In the Kingdom of Saudi Arabia, Khan et al. report that HITs were used in disease surveillance and contact tracing (7). This way, the country improved patient outcomes and minimized the effect of the outbreak.

Given the need to contain the spread of COVID-19 infections and the essential role of sharing data to support decision-making processes as emphasized by crucial global health players, this study aims at determining and evaluating the effect of using health information technologies on managing and responding to the COVID-19 pandemic in Saudi Arabia from health information technology practitioners' perspectives. In addition, this study aims to evaluate the usefulness of applying digital health technology in managing COVID-19, assessing the information exchange capability and information quality provided by HITs, and measuring user satisfaction with HITs.

## 2 Importance of the study and justifications

The study's importance and justification for Saudi Arabia proceed from the fact that the COVID-19 pandemic has sharpened the need for HITs progress in rapid diagnosing and other aspects of healthcare management at hospitals required for saving as many lives as possible. The unprecedented rate of spread has caused the need to change the usual lifestyle to reduce the spread of coronavirus, including minimizing human contact, and HITs help to fulfill this goal by advancing the e-health systems. In general, the application of ITs in healthcare primarily aims to solve the following tasks: electronic registration (no-contact is critical in terms of COVID-19); monitoring and management of the medical care quality; shortening the time for examination and treatment of infected patients; providing consulting medical support; no-contact monitoring of the patient's parameters; providing telemedicine support in the affected areas of Saudi Arabia. In addition, the introduction of information technology in medicine and health care significantly saves the time of medical personnel, which means that it allows employees of medical institutions to devote more time to patients.

It is also worth emphasizing that the need for the study is dictated by clarifying how HITs could upgrade the availability of medical services at hospitals in the face of the pandemic spread. This matter is especially vital for the part of the population located in geographically remote areas and people with disabilities. The whole process of HITs introduction into the healthcare management aims to create a unified medical information space that allows doctors to communicate with each other, refer to the latest technologies' assistance, and interact with the functioning equipment and patients directly from the workplace in real-time. To provide a theoretical reference on how HITs have been used and could be upgraded in Saudi Arabia to respond to the COVID-19 epidemic, this study will review and evaluate the healthcare information technologies that have been applied for managing the outbreak in Saudi Arabia hospitals. The evaluation results will be based on health information technology practitioners' perspectives.

## 3 Research methodology

### 3.1 Purpose of the study

This study aims to determine and evaluate the effect of using health information technologies on managing and responding to the COVID-19 epidemic in Saudi Arabia from health information technology practitioners' perspectives. The research objectives (ROs) of this research are:

1. Evaluating the effect and usefulness of using health information technologies intervention in managing COVID-19.
2. Assessing the Information exchange capability and information quality provided by health information technologies.
3. Measuring the level of user's satisfaction of health information technologies.

### 3.2 The development of the survey instrument

The implemented survey questionnaire for this study consists of three parts as following:

1. The first section contains the demographical data that include gender, age, education level, and occupation.
2. The second section is about the effect of using health information technology interventions to manage COVID-19 that contain rapid diagnosis of patients with COVID-19, test of suspected individual, remote monitoring of patients with COVID-19 isolated at the hospital, home monitoring of infected patients who do not need to be isolated at the hospital, robotic based treatment of the infected patient, Tele-Health consultation, and pharmaceutical delivery.
3. The third section contain the factors as follow:
  A. Perceived usefulness in early diagnosis and isolation of cases of COVID-19, planning of activities regarding COVID-19, providing a better experience without imposing the risks to healthcare and other workers, providing better risk assessment and global public health emergency of this virus, reducing medical errors, promoting a flexible working environment of treatment, improving treatment practices, Improving quality of patients' care, protecting the privacy of patient's information, preventing the spread of COVID-19, reducing the number of waiting list, and saving time.
  B. Information exchange capability: Health information technology provides easy and rapid information exchange (Ex: test results and patient referral information) between medical institutions.
  C. Information quality: always find the needed information, and provided information is easily understood.
  D. Perceived ease of use: Health information technologies are easy to use.
  E. Satisfaction: practitioners' satisfaction with the outcomes of health information technology.

### 3.3 Data collection and analyses

This research adopted a primary analysis and a cross-sectional study design. The data for the study was gathered from health information technology practitioners in the Kingdom of Saudi Arabia. The researchers administered questionnaires as the primary data collection technique. The questionnaire gathered data concerning participants' demographic elements, particularly gender, age, and occupation. In addition, the questionnaire gathered data on digital health technologies intervention used in managing COVID-19, perceived usefulness of using digital health technology in managing COVID-19, information exchange capability, information quality, and perceived ease of use and satisfaction with HITs. The questionnaire was administered via an online survey form. The participants accessed the questionnaire through a web link (URL) and could fill it out at any time or place. Responses to the survey were measured using a 5-point Likert scale.

A total number of 371 participants responded to the survey questionnaire in March 2022. The study's sample size included all Saudi electronic university students who are healthcare practitioners or healthcare administrators. In addition, the study's population comprised other health information technology practitioners from different healthcare organizations in Saudi Arabia. This study adhered to ethical research standards, including obtaining ethical approval from Saudi Electronic University's institutional research board (IRB), participants' consent forms, and other research-related forms and assuring participants of their privacy and confidentiality.

The data collected in the study were analyzed using SPSS statistical software version 26. This data analysis tool extracted the data and performed the descriptive analysis. Since all investigated variables in this study are categorical, the frequency distribution table is used to display the number of occurrences of each variable. Frequency counts, percentages, and bar charts are plotted for all variables to visualize the data.

### 3.4 Validity and reliability

In this research, Cronbach‘s Alpha was calculated for Reliability analysis and Pearson correlation coefficient was used for measuring validity of the dimensions.

According to Table 1, it is notable that the dimensions have excellent reliability where Cronbach‘s Alpha values >0.7 for both dimensions.

**Table 1:** Cronbach‘s alpha for reliability.

**Dimension**	**Number of items**	**Cronbach's Alpha**
Digital health technologies intervention during COVID-19	7	0.781
Perceived usefulness	12	0.966

As showing on Tables 2, 3, all items have high validity where the correlation coefficients between dimension and its items are significant and >0.4.

**Table 2:** Pearson correlation for validity of digital health technologies.

**Items**	**Correlation**
Rapid diagnosis of patients with COVID-19	0.689^**^
Testing of the suspected individual.	0.701^**^
Remote monitoring of patients with COVID-19 isolated at the hospital	0.715^**^
Home monitoring of infected patients who do not need to be isolated at the hospital.	0.655^**^
Robotic based treatment of the infected patient	0.629^**^
Tele-health consultation	0.611^**^
Pharmaceutical delivery.	0.613^**^

^**^Correlation is significant at the 0.01 level (2-tailed).

**Table 3:** Pearson correlation for validity of perceived usefulness.

**Items**	**Correlation**
Early diagnosis and isolation of cases of COVID-19:	0.809^**^
Planning of activities regarding COVID-19:	0.845^**^
Providing a better experience without imposing the risks to workers:	0.856^**^
Providing better risk assessment and global public health emergency of this virus	0.874^**^
Reducing medical errors	0.831^**^
Promoting a flexible working environment of treatment	0.895^**^
Improving treatment practices:	0.911^**^
Improving quality of patients' care:	0.860^**^
Protecting the privacy of patients' information:	0.846^**^
Preventing the spread of COVID-19:	0.835^**^
Reducing the number of waiting lists.	0.843^**^
Saving time.	0.850^**^

^**^Correlation is significant at the 0.01 level (2-tailed).

## 4 Results

This study aimed to determine and evaluate the effect of using health information technologies on managing and responding to the COVID-19 pandemic in Saudi Arabia from health information technology practitioners' perspectives. In addition, the present study evaluated the usefulness of digital health technologies in managing COVID-19, assessed information exchange capability and quality, and measured user's satisfaction with HITs. This section presents the results of the study and integrates the findings with existing literature.

### 4.1 Demographic data

Table 4 presents that most participants were female, constituting 57.4% of the total sample, while males accounted for 42.6% of the respondents. Furthermore, age demographics indicate that 37.7% of respondents fall within the 18–25 years age group, 21.6% are in the 26–30 years age bracket, 19.4% are aged 31–35, 12.4% are between 36–40 years old, and 8.9% are 41 years old or older. In terms of educational qualifications, the majority of respondents hold a bachelor's degree, comprising 75.7% of the sample. Additionally, 14.8% have obtained a Diploma, while 4.9% possess a master's degree. A smaller proportion, 3.2%, have achieved a Doctorate, and 1.3% hold a Professional's degree. Regarding occupation, the study's participants encompass a variety of professional backgrounds. Administrators make up 35.3% of the respondents, while students account for 10.0%. Other healthcare-related roles, including nurses (5.9%), pharmacists (3.0%), physicians (1.9%), and radiologists (1.6%), are also represented. The remaining 42.3% of respondents fall under the category of “Other” occupations, indicating a diverse range of professional backgrounds within the study population.

**Table 4:** Descriptive statistics for demographic data.

**Items**		**Frequency**	**Percent**
Gender	Female	213	57.4
	Male	158	42.6
	Total	371	100.0
Age	18–25	140	37.7
	26–30	80	21.6
	31–35	72	19.4
	36–40	46	12.4
	41+	33	8.9
	Total	371	100.0
Level of education	Bachelor's degree	281	75.7
	Diploma	55	14.8
	Master's degree	18	4.9
	Doctorate	12	3.2
	Professional's degree	5	1.3
	Total	371	100.0
Occupation	Administrator	131	35.3
	Students	37	10.0
	Nurse	22	5.9
	Pharmacist	11	3.0
	Physician	7	1.9
	Radiologist	6	1.6
	Others	157	42.3
	Total	371	100.0

### 4.2 The effect of using health information technologies intervention to manage COVID-19 in Saudi Arabia

**Figure 1:**
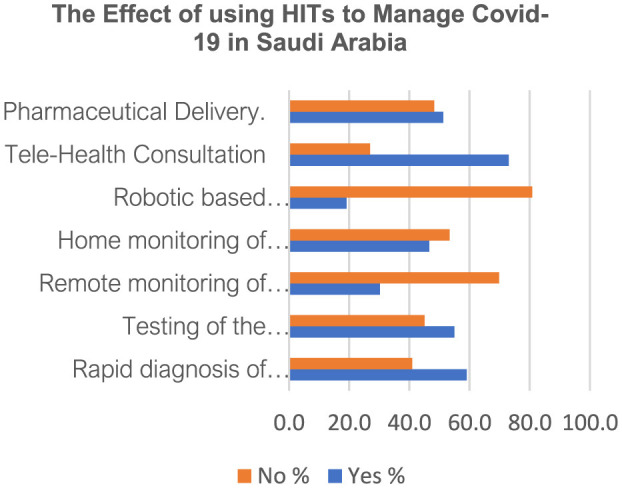
The effect of using health information technologies intervention to manage COVID-19 in Saudi Arabia.

Recently, there has been a strong drive from public health stakeholders, including governments, health agencies, and health systems, to accelerate the adoption of modern technologies in healthcare. The COVID-19 pandemic challenged healthcare stakeholders to adopt innovative approaches to care delivery and protect people and patients from contracting COVID-19 infections. For example, Garfan et al. found that following the outbreak of the COVID-19 pandemic, hospitals implemented telehealth in both urgent and non-urgent medical care, especially in telemonitoring, tele-management, tele-screening, and teleconsulting (8). Figure 1 shows how implementing healthcare technologies in Saudi Arabia influenced COVID-19 management. The current research found that adopting HITs in Saudi Arabia led to telehealth consulting, improved pharmaceutical delivery, and aided screening and monitoring of patients with COVID-19 (Figure 1). This study's results concur with the findings by Khoshrounejad et al., who reported that following the outbreak of the COVID-19 pandemic, telemedicine was widely adopted and applied in screening, triage, prevention, diagnosis, treatment, and follow-up (9). For example, according to the authors, telemedicine helped providers monitor the health status of discharged patients and provide recommendations to manage symptoms in asymptomatic patients (9). Similarly, a study investigating the role of health technologies in managing COVID-19 argued that telehealth systems promote remote consultations, help practitioners manage patients with suspected COVID-19 symptoms remotely, and enable real time monitoring for patients in isolated zones (10, 11). Hence, HITs improve the management of COVID-19.

Implementing health technologies to help mitigate COVID-19 faced significant challenges that hindered the attainment of the desired outcomes. Khoshrounejad et al. identified various barriers, including staff's rejecting the deployed technologies, concerns about the accuracy of subjective patient evaluations, and technical issues (9). For example, the authors mentioned that modern health technologies hindered practitioners from undertaking a comprehensive physical assessment and evaluating vital signs (9). Notably, these challenges, especially technical issues and concerns for the accuracy and effectiveness of telemonitoring and teleconsulting, could be why the respondents indicated that medical innovations in Saudi Arabia did not improve robotic-based treatment of infected persons and remote monitoring of patients with COVID-19 in isolation (Figure 1). For example, Krenitsky et al. argue that on many occasions, patients and practitioners could not connect via the telehealth video interfaces, leading to phone visits that limited providers' capacity to conduct virtual physical exams and gauge patients' general appearance, including other observable vital signs (12). Furthermore, even if patients connected well with their providers via health technologies, Prasad et al. recount that physicians depended on patients' reports of salient symptoms to make a diagnosis, which could lead to reduced diagnostic accuracy (13). Therefore, addressing the many issues that derail the effectiveness of HITs in healthcare is critical for ensuring the provision of quality, safe, and effective patient care.

### 4.3 Perceived usefulness of using digital health technologies intervention in managing COVID-19

Technological innovations in the healthcare industry have emerged as suitable alternatives to patient care approaches and complementary care delivery vehicles. According to James et al., integrating medical technologies into the healthcare industry enhanced patients' access to care (14). In addition, the authors report that apart from increased accessibility, medical technologies save time and contribute to efficiencies, particularly in simple consultations (14). Following the outbreak of the COVID-19 pandemic, Bhatia reported that telehealth aided practitioners with remote access (15). According to the author, this capability was highly beneficial, especially for older adults at a greater risk of infection (15). Similarly, Hoffman noted that medical technologies, such as telehealth, reduced the need for in-person visits to the doctor's office, preventing the spread of COVID-19 infections (16). In addition, the authors reported that telemedicine increased access to care, especially among the underserved communities with limited access to COVID-19-related services (16). Wosik et al. noted that face-to-face interactions between doctors and patients increased the probability of disease transmission and the need to isolate exposed healthcare professionals (17). Thus, apart from increasing access to needed care, medical technologies ensured the safety of at-risk groups, including healthcare experts, children, and persons with underlying conditions.

**Figure 2:**
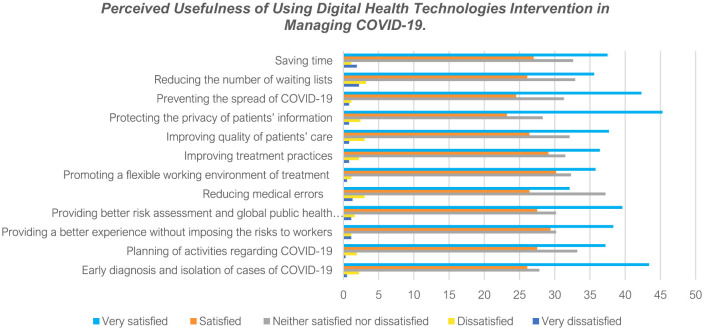
Perceived usefulness of using digital health technologies intervention in managing COVID-19.

Figure 2 above shows healthcare professionals' perceived value of using digital health innovations to manage COVID-19. This study found that deploying digital health technologies helped Saudi Arabia's health care providers to diagnose and isolate COVID-19 cases early, plan COVID-19-related activities, deliver a better experience to patients without imposing COVID-19 risks to health workers, and establish a suitable risk assessment tool to ensure an effective global public health emergency response. In addition, this study reported that health practitioners believed that digital health technologies reduced medical errors, promoted a flexible working environment for treating patients, enhanced treatment modalities, improved patient care, ensured patients' privacy and confidentiality, prevented the spread of COVID-19 infections, reduced the number of waiting lists, and saved time.

The relationship between practitioners' perspectives and the perceived usefulness of digital health technologies in managing COVID-19 was significant as each variable had a *p*-value exceeding 0.8, as shown in Table 1. With the high level of correlation, this study's findings conform to the results by Andrews et al. that digital health technologies helped healthcare experts evaluate patients, provide routine care for patients, and deliver uninterrupted and high-quality patient care (18). Notably, these roles were played remotely, meaning that the technologies enabled clinically table people to stay at home, decreasing physical contact between people and reducing the transmission of COVID-19.

### 4.4 Information exchange capability and information quality provided by health information technologies

**Figure 3:**
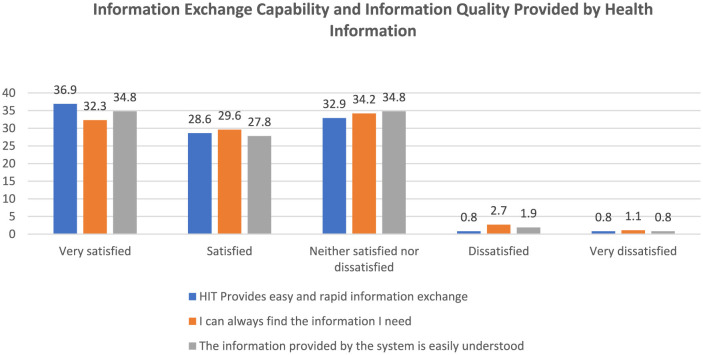
Information exchange capability and information quality provided by health information technologies.

Integrating digital health technologies is a new approach in healthcare, eliciting different opinions from relevant stakeholders, including health professionals. According to Andrews et al., healthcare providers were satisfied with the use of telehealth during the COVID-19 pandemic (18). Similarly, in a study investigating clinicians' perceptions of digital health technologies, Gentry et al. reported that providers were satisfied with the deployment of telehealth, adding that it helped them meet patient needs, preferences, and expectations (19). This outcome is mainly driven by telehealth increasing doctors' access to information. In addition, practitioner training and support are critical in enhancing the effectiveness of digital health technologies (19–22). The current study reports that practitioners could easily find the information they needed and found the information provided by digital health technologies easy to understand and exchange (Figure 3). 65.5% found that HIT provides easy and rapid information exchange and 61.9% of the respondents indicated that they could find the information they needed. Also, 69.6% reported that digital health technologies were easy to understand (Figure 3). This finding indicates that providers in Saudi Arabia were satisfied with the deployment of medical innovations during the COVID-19 pandemic.

### 4.5 Perceived ease of use and the level of user satisfaction with health information technologies

#### 4.5.1 Perceived ease of use

**Figure 4:**
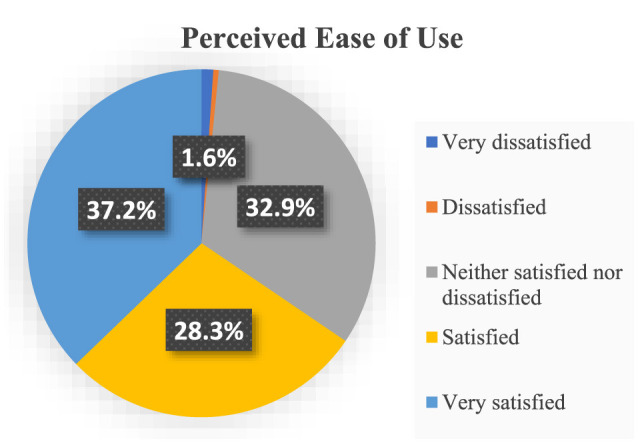
Descriptive statistics for perceived ease of use.

The study results reveal interesting findings concerning the satisfaction levels of respondents regarding the ease of use of health information technologies, as depicted in Figure 4. Notably, a significant portion, representing 37.2% of the participants, expressed a high level of satisfaction, indicating that they were “very satisfied” with the ease of use of these technologies.

Additionally, a substantial 28.3% of respondents reported being “satisfied,” further underlining a positive sentiment toward the usability of health information technologies. Contrasting this, 32.9% of participants fell into the category of “neither,” suggesting a neutral stance or mixed feelings regarding the ease of use. This diversity of responses provides valuable insight into the varying perceptions and experiences of individuals within the surveyed population in relation to health information technologies.

#### 4.5.2 User satisfaction

**Figure 5:**
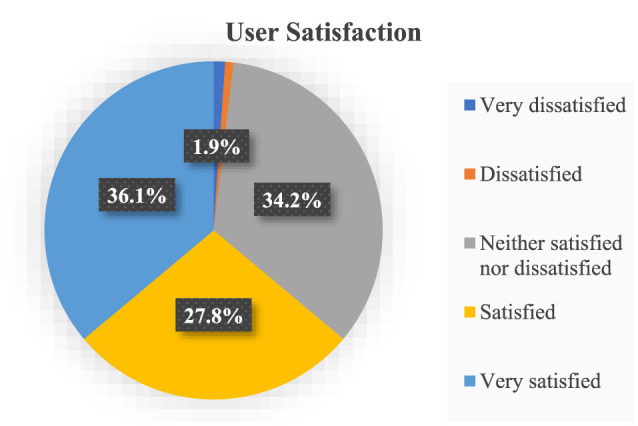
Descriptive statistics for user satisfaction.

The data depicted in Figure 5 highlights a noteworthy trend among the study respondents regarding their satisfaction with the outcomes of utilizing health information technology. A substantial 36.1% of the participants expressed a high degree of contentment, categorizing themselves as “very satisfied” with the results achieved using health information technology.

Moreover, a notable 27.8% of respondents indicated their satisfaction, signifying that they were “satisfied” with the outcomes of employing this technology in their healthcare context. In contrast, a significant segment, comprising 34.2% of the respondents, fell into the category of “neither satisfied nor dissatisfied,” implying a nuanced perspective or a level of ambivalence regarding the outcomes experienced. This diversity in satisfaction levels underscores the complexity of assessing the impact of health information technology, suggesting that individuals' perceptions and experiences vary widely within the surveyed population.

Further analysis and exploration of these sentiments may provide valuable insights into the broader implications and potential areas for improvement in the adoption of health information technology in healthcare settings.

## 5 Conclusion

The current study used primary analysis and a cross-sectional design to investigate the effects of using HITs to manage the COVID-19 pandemic in the Kingdom of Saudi Arabia. Data for the study was collected from HIT practitioners' perspectives using survey questionnaires. The analysis of the data revealed that digital health technologies significantly influenced how Saudi Arabia's health systems managed and responded to the COVID-19 pandemic. In particular, this study reported that the deployment of digital health technologies in Saudi Arabia improved pharmaceutical delivery, enhanced teleconsulting, and facilitated screening and monitoring of COVID-19 patients. However, several areas related to the management of COVID-19 lagged. This study found that implementing digital health technologies in Saudi Arabia did not enhance robotic-based treatment of infected individuals and remote monitoring of isolated persons with COVID-19. The current study associated these adverse outcomes with HIT challenges, including technical issues that limited providers' capacity to conduct virtual physical exams and gauge patients' general appearance and other observable vital signs. Thus, digital health technologies have the potential to improve the management of COVID-19 and future pandemics, but necessary measures must be implemented to manage associated limitations to achieve the desired outcomes.

This study also found that HITs were useful in facilitating interventions for managing the COVID-19 pandemic. Medical technologies improved early diagnosis and isolation of COVID-19 cases, enhanced the planning of COVID-19-related activities, and improved patients' care experiences while ensuring frontline workers' safety. In addition, the study discovered that digital health technologies aided risk assessment, reduced medical costs, created a flexible working environment for providers, improved treatment options, enhanced patient care quality, promoted patients' privacy and confidentiality, saved time, and controlled the spread of COVID-19 infections. The correlation between these variables and the providers' perspectives was high (*p* above 0.8). This study associated these outcomes with the fact that HITs allowed clinically stable people to stay at home, reducing overcrowding and physical contact that, in turn, reduced the transmission of COVID-19 infections. Furthermore, this study found that providers in Saudi Arabia were satisfied with the implementation of digital health technologies. This study reported that the practitioners could easily find the information they needed, and the information obtained from HITs was easy to understand. Therefore, digital health technologies add value to health systems, allowing the implementation of helpful medical interventions and improving providers' satisfaction.

## Data Availability

The raw data supporting the conclusions of this article will be made available by the authors, without undue reservation.
